# Comparison of nine different commercially available molecular assays for detection of SARS-CoV-2 RNA

**DOI:** 10.1007/s10096-021-04159-9

**Published:** 2021-01-29

**Authors:** Ute Eberle, Clara Wimmer, Ingrid Huber, Antonie Neubauer-Juric, Giuseppe Valenza, Nikolaus Ackermann, Andreas Sing, for the Bavarian SARS-CoV-2-Public Health Laboratory Team, Armin Baiker, Bernadett Bartha-Dima, Katja Bengs, Anja Berger, Kerstin Boll, Anja Carl, Jürgen Christian, Alexandra Dangel, Juliana Drdlicek, David Eisenberger, Volker Fingerle, Lars Gerdes, Ottmar Goerlich, Patrick Gürtler, Sabrina Hepner, Bernhard Hobmaier, Christine Hupfer, Regina Konrad, Sandra Lampl, Bernhard Liebl, Gaia Lupoli, Gabriele Margos, Roswitha Müller, Silke Nickel, Mercy Okeyo, Melanie Pavlovic, Sven Pecoraro, Isabel Sahm, Melanie Schauer, Anika Schülein, Eva-Maria Schürmann, Gesine Schulze, Nelly Scuda, Stefanie Singer, Thorsten Stellberger, Bianca Treis, Christian Tuschak, Pia Zimmermann

**Affiliations:** 1 Unit of Virology, Bavarian Health and Food Safety Authority, Oberschleißheim, Germany; 2 Public Health Microbiology Unit, Bavarian Health and Food Safety Authority, Oberschleißheim, Germany; 3 Molecular Biology Unit, Bavarian Health and Food Safety Authority, Oberschleißheim, Germany; 4 Unit of Veterinary Virology, Bavarian Health and Food Safety Authority, Oberschleißheim, Germany; 5 Unit of Hospital Hygiene, Bavarian Health and Food Safety Authority, Erlangen, Germany; 6 Ludwig Maximilians-Universität München, Munich, Germany

**Keywords:** SARS-CoV-2, Molecular testing, Laboratory diagnostics, Real-time PCR, COVID-19, Coronavirus, Infectious disease, Diagnostics

## Abstract

To face the COVID-19 pandemic, the need for fast and reliable diagnostic assays for the detection of SARS-CoV-2 is immense. We describe our laboratory experiences evaluating nine commercially available real-time RT-PCR assays. We found that assays differed considerably in performance and validation before routine use is mandatory.

The localized outbreak in the province Hubei and surrounding areas in China at the end of December 2019 led to a rapid spread throughout the world increasing numbers in cases and deaths. On March 11, 2020, WHO declared COVID-19 a pandemic. Major strategies to face this pandemic are (i) to minimize social contacts, (ii) to test people suspected for COVID-19, (iii) to perform strict containment measures for SARS-CoV-2 (severe acute respiratory syndrome coronavirus 2)-infected persons, and (iv) to conduct thorough contact tracing. Public health authorities and other diagnostic laboratories are in urgent need for diagnostic tools, which are fast and reliable. The rapid pandemic spread resulted in various shortages not only in safety equipment, but also in diagnostic reagents and supply. Due to imminent problems with limited test resources, a foresighted and dynamic lab strategy is needed to avoid possible shortages in lab supplies including test kits. An important pillar of the strategy chosen by the Public Health Microbiology laboratory at the Bavarian Health and Food Safety Authority (LGL), Germany, was the pro-active decision to diversify diagnostic tools. Moreover, our laboratory was confronted with an increasing number of various test kits offered by very different companies but of unclear quality standards.

In this paper, we describe our laboratory experiences comparing nine commercially available real-time RT-PCR assays, using a Bio-Rad CFX 96 cycler.

## Routine molecular diagnostics for SARS-CoV-2 in a public health laboratory

**Fig. 1:**
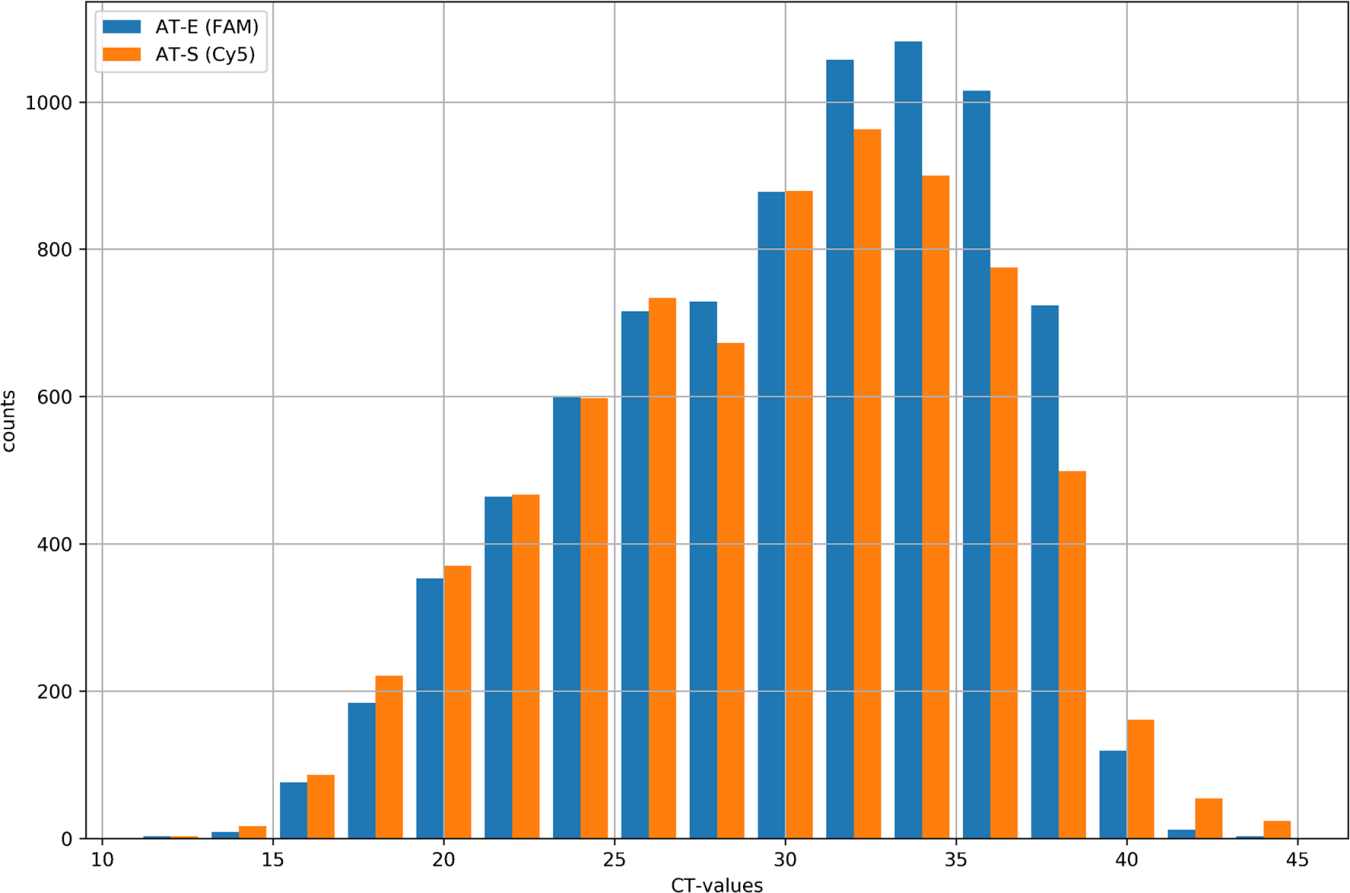
Analyses of SARS-CoV-positive samples (*n* = 8206) over 14 weeks from February to May at the LGL: the histogram gives, Ct values after amplification within the E-Gen (AT-E) (blue, detection of B lineage betacoronavirus, *n* = 8021, 9.47% of positive samples only E gene positive) and the S-Gen(AT-S) (red, detection of SARS-CoV-2, *n* = 7425, 2.25% of positive samples only S gene positive), RealStar SARS-CoV-2 RT-PCR Kit RUO, Altona Diagnostics against the number of samples. COVID, coronavirus disease; LGL, Bavarian Health and Food Safety Authority

Based on an initial evaluation of SARS-CoV-2-RT-PCR assays available at the very beginning of the pandemic in Germany [1], we established the RealStar SARS-CoV-2 RT-PCR Kit RUO, Altona Diagnostics on the Bio-Rad CFX96 Touch Real-Time PCR Detection System (Bio-Rad, Feldkirchen, Germany) as routine method for SARS-CoV-2 diagnosis. Between the calendar weeks 5/2020 and 19/2020, approximately 70,000 mainly respiratory samples were analyzed at the LGL. Eight thousand two hundred six thereof were diagnosed as SARS-CoV RNA positive (Fig. 1). The assay amplifies sequences of the E gene of B lineage betacoronaviruses and of the S gene specific for SARS-CoV-2 that are detected with fluorophores/Fam and Cy5, respectively.

## Comparison of nine commercially available molecular diagnostic kits for SARS-CoV-2

To compare the performance of further eight PCR kits for SARS-CoV-2 diagnostic, field samples as well as SARS-CoV-2 reference virus genomes in defined genome copy number and genomes of putatively contaminating agents were used. Respiratory samples, mainly nasopharyngeal or pharyngeal swabs were obtained from patients and contact persons (see column “sample size” in Table 1). To increase the total sample volume for further testing with the RealStar SARS-CoV-2 RT-PCR Kit RUO, Altona Diagnostics pretested positive and negative samples were diluted 2 to 3 fold with NaCl 0.9%. Control material (positive controls) comprising Human 2019-nCoV RNA (reference number 026N-03889) and SARS-CoV Frankfurt 1 RNA (reference number 004N-02005) was ordered from the European Virus Archive (EVAg) [2]. Cross-reactivity was tested for human coronavirus HCoV 229E^1^, human coronavirus HCoV-OC43^1^, human coronavirus HCoV-NL63^1^, MERS-CoV^1^, human rhinovirus type 30^1^ and type 49^2^, 2 samples human metapneumovirus Typ A^1,2^, Parainfluenzavirus type 2^2^ and 3^1^, Influenzavirus (A(H1N1)pdm09, A(H3N2), B Yamagata- and Victoria-Lineage)^1^, respiratory syncytial virus A^1^ and B^1^, Adenovirus type 11^2^, *Bordetella pertussis*^*2*^, *Chlamydophila pneumoniae*^*1*^, and *Mycoplasma pneumoniae*^*1*^. Respective reference samples were obtained from INSTAND e.V., Düsseldorf, Germany. (Footnotes: ^1^pathogen panel 1, analysed with all mentioned tests; ^2^pathogen panel 2, analysed with AT, MG, G, FTD, SG, and WB (abbreviations see Table 1)) As expected, analysis of SARS-CoV-1 yielded positive results for lineage B-betacoronavirus (B-βCoV) detection systems and negative results for with detection systems for SARS-CoV-2-specific genetic regions. Sample sizes are given in Table 1 column “sample size.” The variation in sample sizes between different assays is due to the different amounts of available PCR reaction.

**Table 1:** Comparison of different commercial real-time RT-PCR systems for SARS-CoV-2 RNA detection

Company (site)	Used abbreviation	Target gene (fluorophore)	Specific for SARS-CoV-201	Number of replicates^b^	42PCR efficiency (%)^a,b^, linearity (R2)^b^	Limit of detection (copies/reaction)^b^	Sample size positive/negative	Number of TP	Number of FP	Number of TN	Number of FN	Median Ct	Sensitivity (%)	Specificity (%)	Run time (hours)
Altona Diagnostics (Hamburg, Germany)	AT	AT-E (FAM)	No	4	95.45/0.9931	10	39/25	35	0	25	4	28.07	90	100	02:15
		AT-S (Cy5)	Yes	4	105.43/0.9956	100	35/29	34	0	29	1	28.35	97	100	
Mikrogen Diagnostik (Neuried, Germany)	MG	MG-E (FAM)	No	4	98.97/0.9924	10	39/25	38	0	25	1	29.74	97	100	01:32
		MG-ORF1a (Hex)	Yes	4	105.82/0.9943	10	37/27	36	0	27	1	29.88	97	100	
gerbion GmbH & Co KG (Kornwestheim, Germany)	G	G-RdRP (FAM)	Yes	2	ND	6000	35/29	17	0	29	18	28.24	49	100	01:32
		G-E (Cy5)	No	2	76.56/0.9896	60	39/25	32	0	25	7	29.21	82	100	
Primerdesign Ltd. (Camberley, UK)	PD	PD-nCoV (FAM)	Yes	2	87.30/0.9969	80	23/18	20	0	18	3	33.26	87	100	01:44
Fast Track Diagnostics (Esch-sur-Alzezze, Luxembourg)	FTD	FTD-N/Orf1ab (FAM)	Yes	4	91.13/0.9929	100	38/26	38	0	26	0	29.57	100	100	01:37
Shanghai Fosun Long March Medical Science Co., Ltd. (Shanghai, China)	SF	SF-Orf1ab (FAM)	Yes	2	95.77/0.9999	100	17/16	16	0	16	1	34.68	94	100	01:39
		SF-N (HEX)	Yes	2	95.77/0.9999	10	16/17	16	0	17	0	34.00	100	100	
		SF-E (ROX)	Yes	2	120.04/0.9692	10	17/16	16	0	16	1	35.13	94	100	
SolGent Co., Ltd. (Daejeon, Korea)	SG	SG-N (FAM)	Yes	2	87.13/0.9900	50	37/27	36	0	27	1	30.22	97	100	01:55
		SG-Orf1a (VIC)	Yes	2	89.81/0.9757	50	37/27	34	0	27	3	29.42	92	100	
WELLS BIO, INC. (Gangseo-gu, Republic of Korea)	WB	WB-RdRPP2 (FAM)	Yes	2	89.81/0.9757	100	35/29	23	0	29	12	24.08	66	100	01:29
		WB-E (FAM)	No	2	71.84/0.9747	10	39/25	24	0	25	15	23.70	62	100	
BGI (Wuhan, China)	BGI	BGI-nCoV (FAM)	Yes	2	98.51/0.9946	10	22/16	21	0	16	1	32.20	95	100	01:42

*ND* not determined, *TP* true positive, *FP* false positive, *TN* true negative, *FN* false negative

^a^*E* = 10^−1/slope^ − 1

^b^Detection limit, linearity, and PCR efficiency were determined using Human 2019-nCoV RNA (reference number 026N-03889) from the European Virus Archive (EVAg) with replicates, as given in column “number of replicates.” 10-fold serial dilutions were performed

RNA was extracted from samples using the QIAamp Bio Robot kit (QIAGEN) on a Hamilton Microlab Star (Hamilton, Bonaduz, Switzerland). Extractions were performed in triplicates to obtain sufficient elution volume to execute all PCR assays with identical sample preparations. Eluates were pooled and stored in 30–50-μl aliquots at − 80 °C before carrying out PCR assays. All PCR assays were performed according to the instructions of the respective manufacturers. During the evaluation phase, the threshold was manually set to 150 RFU (Bio-Rad CFX96 Touch Real-Time PCR Detection System) irrespective of the assay validated and the fluorophore. Amplification plots with exponential curve increases were considered positive.

A summary of the results and features of the nine commercially available PCR assays tested is shown in detail in Table 1.

The limit of detection was determined using Human 2019-nCoV RNA (reference number 026N-03889) from the European Virus Archive (EVAg) containing defined genome copy numbers. The PCR assays of Altona Diagnostics (AT), Mikrogen Diagnostik (MG), Shanghai Fosun Long March Medical Science Co (SF), and BGI reached a detection limit of about 10 copies/reaction (see column “limit of detection” in Table 1). Notably, due to the limited amount of PCR reagents available for testing, the number of achievable replicates was limited to two–four (Table 1), preventing detailed statistical analyses. Repetition of these analyses using more replicates is recommended before clinical use.

The sensitivity of the assays was determined dividing the number of tested true-positive sample with the total number of pretested positive samples. Applying this procedure, the assays of Fast Track Diagnostics (100%, FTD), Mikrogen Diagnostik (97%, MG), SolGent Co., Ltd. (97%/92%, SG-N/SG-Orf1a), Altona Diagnostics (90%/97%, AT-E/AT-S), BGI (95%), and Shanghai Fosun Long March Medical Science Co., Ltd. (94%, SF) showed decreased sensitivities according to the order mentioned. The tests from gerbion GmbH & Co KG (G-RdRP), WELLS BIO, INC. (WB-RdRPP2), and Primerdesign Ltd. (PD) showed the lowest values of 49%, 62%, and 86%, respectively (see columns “number of FN” and “sensitivity” in Table 1).

The specificity of each assay was tested in order to discriminate SARS-CoV-2 signals from other respiratory pathogens such as other coronaviruses. All assays gave no false-positive test results (see columns “specificity” and “number of FP” in Table 1).

Due to the limited amount of reagents available for testing sensitivity and specificity performances of BGI, Shanghai Fosun Long March Medical Science Co., Ltd. (SF), and Primerdesign Ltd (PD) test kits, results should be interpreted with caution (see column “sample size” in Table 1).

**Fig. 2:**
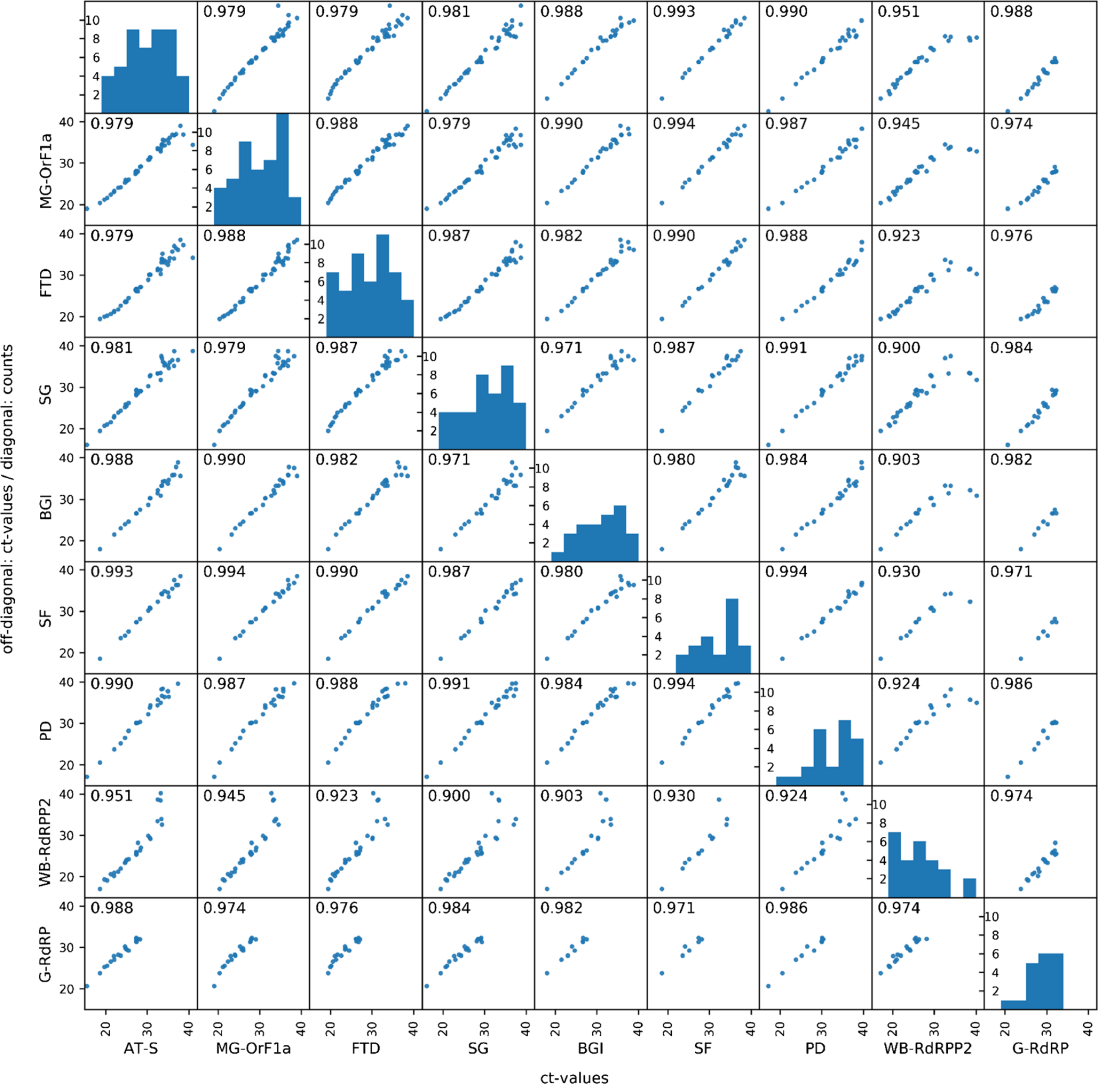
Multiple correlation of different assays (abbreviation see Table 1). Diagonal shows the distribution of Ct values of positive tested samples in histogram. Numbers represent the Pearson standard correlation coefficient (*R*)

Additionally, a multi-correlation analysis was conducted focusing on the specific results for SARS-CoV-2. For assays showing more than one specific result, the smallest Ct value was included in the analysis. Most tests showed Pearson standard correlation coefficients above 95%. The distribution of Ct values is shown in the histograms in Fig. 2 (diagonal). The Ct values of Primerdesign Ltd. (PD) shifted to higher Ct values leading to a higher median Ct value of 33.26. On the other hand, gerbion GmbH & Co KG (G-RdRP) and WELLS BIO, INC. (WB-RdRPP2) median Ct values were 28.24 and 24.08, respectively, while higher Ct values above 32.04 and 33.43 were not routinely detected (see Fig. 2 and Table 1).

## Discussion

A main task for public health laboratories is to deliver reliable results for emerging pathogens of global threat to prevent spread. Since on January 7, 2020, a novel coronavirus was identified, and shortly after, the first sequence of the new strain was published. Shortly afterwards [3, 4], our laboratory faced the difficult task to decide on appropriate diagnostic assays. Due to an enormous demand for diagnostic reagents, we were confronted with a shortage of PCR kits to detect SARS-Cov-2. Furthermore, we had to deal with offers for PCR kits from different origins and of unclear quality standards.

To decide which of the kits available on the market at the time would be best for our diagnostics, we tested a number of kits that had given promising results in other studies [5] and targeted two or more SARS-CoV-2 genes in one PCR setup. This was the case in all but one commercial kit (WELLS BIO, INC., WB). For two of the kits (BGI and Primerdesign (PD)), it was not possible to retrieve information on how many genes were targeted and which genetic regions were amplified. In our view, the detection of more than one gene seemed crucial as different target genes may differ in specificity and sensitivity. In our hands, the kits of Fast Track Diagnostics (FTD), Mikrogen Diagnostik (MG), Altona Diagnostics (AT), and Shanghai Fosun Long March Medical Science Co., Ltd. (SF) gave the best results. Since for all assays, identical extracted material was used, we assume that the observed differences between kits are due to differences in targeted genome regions and/or primer design. Further complications were due to the fact that the kits of BGI, WELLS BIO, INC., WB, and SolGent Co., Ltd. contained the internal control as part of the Mastermix and could not be used as an extraction control resulting in lack of a control system for the extraction procedure.

In outbreak situations, it is crucial to efficiently optimize all workflows even with limited resources of human workforce, reagents, and devices.

Comparable results were published for Altona Diagnostics, Mikrogen, and BGI in other studies [5].

## Conclusion

The quality of the tested PCR assays showed very different results. Therefore, it is very important to control commercially available kits for their performance characteristics prior to use in routine laboratory diagnosis. Even in situations with enormous pressure due to reagent shortage, quality must not be neglected. Fast and reliable results are crucial dealing with the COVID-19 pandemic. The Fast Track Diagnostics (FTD), Mikrogen Diagnostik (MG), Altona Diagnostics (AT), and Shanghai Fosun Long March Medical Science Co., Ltd. (SF) outperformed all other tested mentioned above. In addition to correct results, it is recommended to use assays with at least two different target regions either in one channel or two to have a more robust assay for a fast evolving pathogen.
